# Influence of Sodium Salicylate on Self-Aggregation and Caffeine Solubility in Water—A New Hypothesis from Experimental and Computational Data

**DOI:** 10.3390/pharmaceutics14112304

**Published:** 2022-10-26

**Authors:** Milan Vraneš, Teona Teodora Borović, Patrik Drid, Tatjana Trivić, Renato Tomaš, Nenad Janković

**Affiliations:** 1 Faculty of Sciences, University of Novi Sad, Trg Dositeja Obradovića 3, 21000 Novi Sad, Serbia; 2 Faculty of Sport and Physical Education, University of Novi Sad, Lovćenska 16, 21000 Novi Sad, Serbia; 3 Faculty of Chemistry and Technology, University of Split, Ruđera Boškovića 35, HR-21000 Split, Croatia; 4 Department of Chemistry, Faculty of Science, University of Kragujevac, Radoja Domanovića 12, 34000 Kragujevac, Serbia

**Keywords:** caffeine, sodium salicylate, hydration, self-aggregation, solubility

## Abstract

The present study analyzed experimental data from volumetric and viscosimetric measurements and computational simulations to understand caffeine hydration and aggregation properties in 0.1 mol∙kg^−1^ of sodium salicylate aqueous solution. Sodium salicylate reduces the bitter taste and increases the solubility of caffeine in water, which is the main reason for their combination in food products. The results noted in volumetric and viscosimetric measurements indicate that sodium salicylate promotes the self-aggregation of caffeine in water. After self-aggregation, the hydration number of caffeine significantly increases. Molecular simulations have allowed us to hypothesize how salicylate increases caffeine solubility. At the molecular level, relocating salicylate moiety from the parallel stacking (π–π) aromatic complex with caffeine and its hydration could be the main reason for increasing the solubility of caffeine in water. The presented study provides clear guidelines on the choice of additives to increase caffeine’s solubility in aqueous media. The choice of salicylate as an additive to increase the solubility of caffeine is very important because caffeine and salicylate are found in combination in a large number of formulations.

## 1. Introduction

**Figure 1:**
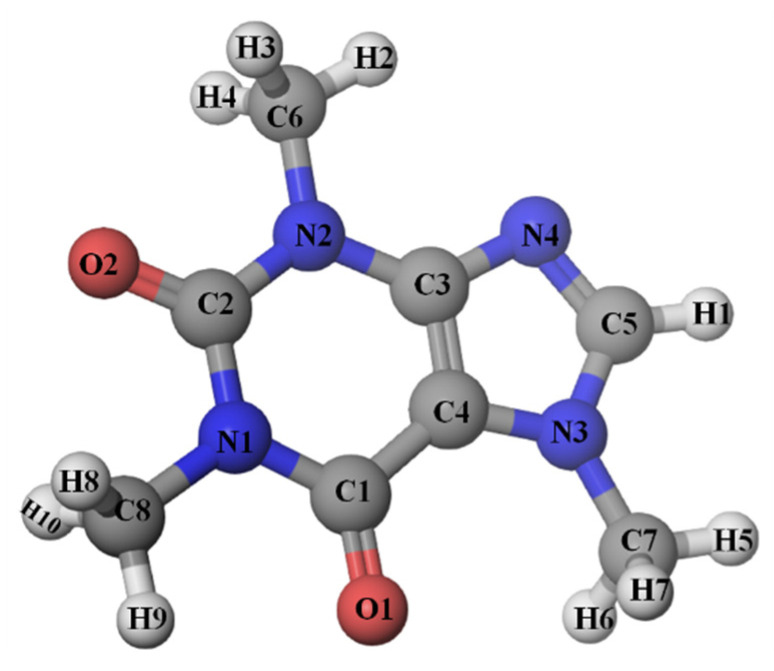
Structure of caffeine molecule and atom-numbering scheme.

Caffeine (1,3,7-trimethylxanthine, see Figure 1) belongs to the group of xanthine alkaloids together with theophylline and theobromine [1]. As an ingredient in coffee, black and green tea, and soft and energy drinks, caffeine is the most widely used psychoactive substance [2]. Pure caffeine is a solid substance, odorless and slightly bitter-tasting. The important physiological effects of caffeine are stimulation of the central nervous system, gastric acid secretion, and blood pressure increase in the short term of action [3]. The solubility of caffeine in water is relatively low (approximately 16 mg·mL^−1^ at room temperature), which is one of the crucial problems, especially in preparations consumed or stored at low temperatures [4]. The hydrophobicity of pharmacologically active drugs and active ingredients of supplements is a major problem in drug design, food science, delivery, drug effect, and bioavailability [5]. The possibility of transport and achieving optimal concentration in biological fluids is limited due to their low solubility in water.

It is considered that caffeine has limited solubility in water due to the self-association and aggregation of caffeine molecules by hydrophobic interactions [6,7,8,9]. A common solution for increasing solubility and limiting aggregation is to add some biocompatible molecules, excipients, or hydrotropes. Ahmad et al. investigated different salts’ (Na_2_SO_4_, NaCl, NaClO_4_, and NaSCN) effects on caffeine solubility and self-association, of which NaClO_4_ and NaSCN increase the solubility of caffeine in water [7]. The same effects were investigated by Shumilin et al. using sugars [5]. Mejri et al. used sucrose and β-cyclodextrin to determine hydration and self-association of caffeine molecules in an aqueous solution [10]. Cui explores interactions of a system consisting of caffeine, riboflavin, and water [11]. Vraneš et al. analyzed caffeine hydration and aggregation properties in aqueous solutions and compared these results to caffeine properties in the presence of adenosine triphosphate (ATP) molecules. They concluded that ATP negatively affects caffeine due to self-aggregation, leading to reduced bioavailability in the biological systems [12].

**Figure 2:**
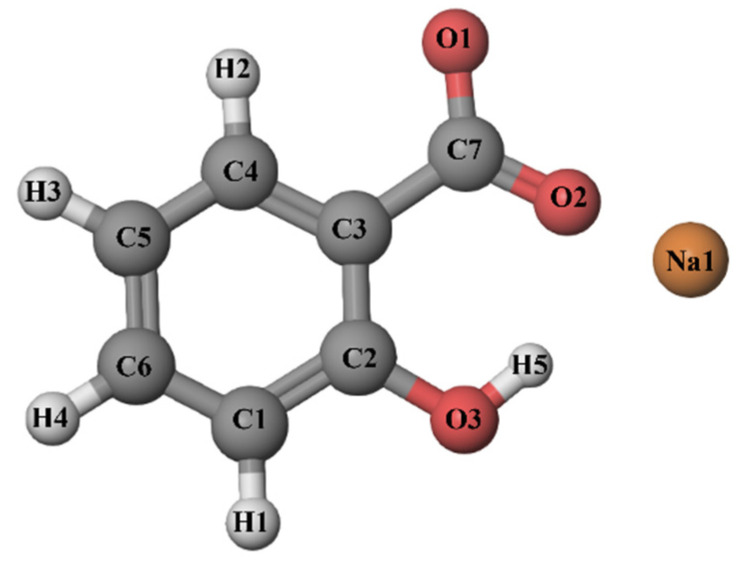
Structure of sodium salicylate molecule and atom-numbering scheme.

In earlier works, interactions between caffeine and benzoic acid derivatives (sodium benzoate, sodium salicylate, salicylic acid, acetylsalicylic acid, etc.) [13,14,15] were investigated, but how these benzoic acid derivatives affect the better solubility of caffeine has not been investigated so far. The mechanism by which these compounds increase the solubility of caffeine in water has remained unknown. The influence of sodium salicylate on the solubility of caffeine has not yet been experimentally determined. Sodium salicylate (Figure 2) (SS) is a non-steroidal anti-inflammatory drug, and it belongs to the derivates of salicylic acid and is used as an analgesic, antipyretic, antioxidant, and antimicrobial drug [16]. In spite of that, salicylate is most frequently used in the food industry as a dietary supplement and preservative. It can also be found in food as a naturally occurring compound [17,18,19].

Ankita et al. analyzed physicochemical properties of sodium salicylate in aqueous solution and aqueous glucose/sucrose solutions at temperatures from 293.15 to 318.15 K. They concluded that sodium salicylate is a typical structure-maker (positive values of *B*-coefficient which decrease with increasing temperature) [20]. Drugs containing a combination of caffeine and acetylsalicylic acid have been available for many years and are widely used clinically [21]. It has been proven that the addition of caffeine to drugs containing acetylsalicylic acid leads to increased activity of the drug; for example, the onset of analgesia is accelerated, and pain relief is more effective compared to drugs that do not contain caffeine in their composition [22]. Caffeine in combination with SS can be found in various drugs or preparations, such as Algopirin (used for acute low back pain), Acetaminophen (used for temporary relief of pain), Excedrin (used for Migraine headache), Ephedrine-Caffeine-Acetylsalicylate stack (drug combination used in weight loss), etc. Today, there is an increasing number of scientific papers that research the influence of sodium salicylate molecules on caffeine, both in drug formulations and aqueous solutions. For example, one of the recent works by Kim et al. talks about the influence of caffeine on the absorption rate of aspirin [23]. Therefore, our goal in this paper is to discuss how adding a typical structure-maker in 0.1 mol·kg^−1^ of SS affects caffeine hydration and aggregation properties through hydration parameters obtained from volumetric and viscosimetric measurements, solubility determination, and computational simulations.

## 2. Materials and Methods

### 2.1. Materials

Caffeine (anhydrous, CAS number: 58-08-2, mass fraction purity ≥ 99%), chloroform (CAS number: 67-66-3, mass fraction purity ≥ 99%), and sodium salicylate (CAS number: 54-21-7, mass fraction purity ≥ 99,5%), presented in Table S1, were procured from Sigma Aldrich (St. Louis, MI, USA), and were used as-received without further purification.

### 2.2. Apparatus and Procedure

The 0.1 mol∙kg^−1^ SS aqueous solution was used as a solvent to prepare a set of caffeine solutions for density and viscosity measurements up to caffeine molality of *m* = 0.10584 mol·kg^−1^. Experimental measurements of solution densities and viscosities were performed at five temperatures: *T* = 293.15, 298.15, 303.15, 308.15, and 313.15 K. A detailed description of the apparatuses used is provided in the Supplementary Materials.

The determination of caffeine solubility in pure water was performed at five temperatures: *T* = 293.15, 298.15, 303.15, 308.15, and 313.15 K. Solubility of caffeine in pure water was determined using the gravimetric method already described in the work of Romero and Oviedo [24]. A double-layer glass flask with a volume of 60 cm^3^ was used for solubility determination. It was held in a constant temperature bath for the necessary time to reach equilibrium at each temperature. The temperature was controlled with a Lauda E-100 circulator. The flask was placed on an automatic mixer with a magnetic core. Caffeine saturated solutions were prepared by adding an appropriate amount of caffeine to 40 cm^3^ of ultrapure water and stirring the flasks containing the samples with a temperature control of ±0.01 K. Before transferring the samples, the syringe with a needle was immersed in the flask, and the stirrer was turned off for 2 h to allow the undissolved solute to settle.

The samples of 5 cm^3^ were withdrawn using a syringe. The needle was quickly removed, and a filter (33 mm diameter sterile Millex-HA syringe filter with a 0.45 µm pore size mixed cellulose esters membrane) was placed on the syringe to prevent undissolved caffeine from causing inaccurate results. The samples were transferred to 5 cm^3^ pre-weighed glass flasks and their mass was measured with an accuracy of ±1 × 10^−5^ g in the lower range. The samples were placed in a vacuum oven at 383.15 K and evaporated to dryness to recover the caffeine. The mass of the caffeine was determined gravimetrically. Solubility measurements were performed at temperatures between 293.15 and 313.15 K. Each obtained value represents the average of at least five independent measurements. The uncertainty in the mass fraction solubility is ±0.001. Reproducibility was found to be better than ±0.002.

The determination of caffeine solubility in 0.1 mol∙kg^−1^ SS aqueous solutions was performed at the same temperature range, glass flask, and conditions as in the previously described method. However, in the presence of sodium salicylate, it is necessary to separate the caffeine. The separation of caffeine and sodium salicylate molecules was performed by liquid–liquid extraction with chloroform. Caffeine dissolves well in chloroform, while the solubility of sodium salicylate is negligible. Due to that, the procedure has been slightly modified. After taking a 5 cm^3^ sample from the double-layer glass flask, the sample was transferred to a glass separating funnel, and 10 cm^3^ of chloroform was added. Extraction with chloroform was repeated three times for each sample. After each extraction, the bottom chloroform layer was dropped into a previously measured balloon. Chloroform was removed using a rotary evaporator. The sample was further dried for 2 h at 383.15 K until a constant mass was reached. The extracted mass of caffeine corresponds to the mass of caffeine that can be dissolved in a sampled volume of 0.1 mol·kg^−1^ of SS aqueous solution at a given temperature.

The interactions of caffeine in pure water and in SS aqueous solution were theoretically investigated with molecular dynamics (MD) simulations using the Yasara Structure (version 16.3.8.) [25] packages by opting for the use of AMBER14 as a force field. For AMBER14, the TIP3P water model was used. These MD simulations were accomplished in NPT canonical ensemble at a constant pressure of 1 × 10^5^ Pa and a temperature of 298.15 K using periodic boundary conditions. Pressure and temperature control was performed using a Nose–Hoover barostat, while the “cut-off” radius used for all Lenard–Jones interactions was 10 Å. To imitate physiological conditions, counter-ions were added to neutralize the system. Na or Cl was added in replacement of water to give a total NaCl concentration of 0.9%. pH was maintained at 7.4. The particle-mash Ewald (PME) summation was applied to correct the effect of the cut-off radius with respect to Coulomb interactions. The initial phase of the MD simulation involved establishing equilibrium, which lasted an average of about 10 ns, and these data were not analyzed later. The system after equilibration was analyzed by examining the trajectory from which the radial distribution functions (RDF) were examined. All simulations lasted a minimum of 60 ns.

## 3. Results and Discussion

### 3.1. Solubility Results

Experimental data for the solubility of caffeine in 0.1 mol∙kg^−^^1^ SS aqueous solutions and the solubility of caffeine in pure water were obtained experimentally and presented in Table 1. As can be seen, the solubility of caffeine increased with temperature in both cases. In 0.1 mol∙kg^−^^1^ SS aqueous solutions, caffeine had a 2–2.7 times higher solubility than caffeine in pure water, depending on the temperature.

**Figure 3:**
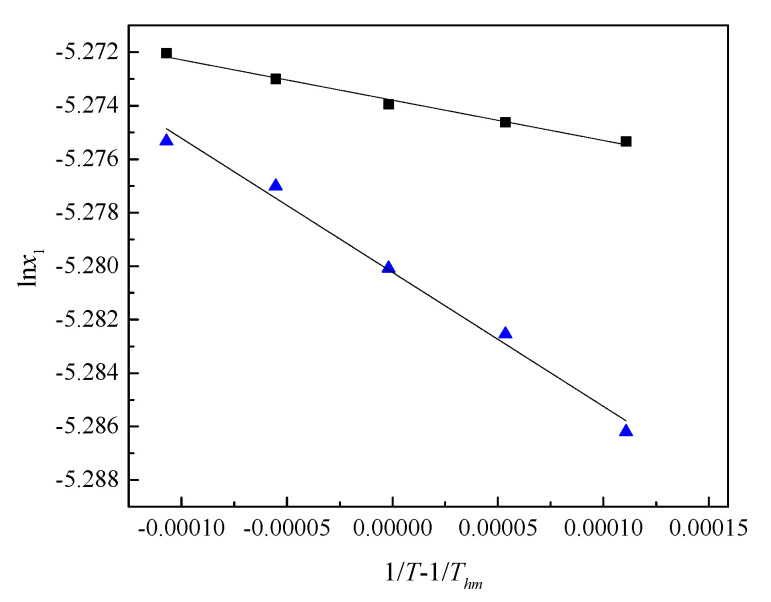
The plot of the natural logarithm of the mole fraction of (■) caffeine in a saturated 0.1 mol∙kg^−1^ SS aqueous solution and (▲) caffeine in a saturated aqueous solution, ln*x*_1_ versus 1*/T* − 1*/T*_hm_.

Based on the modified Van ’t Hoff equation, the apparent standard dissolution enthalpy (Δ_sol_*H*°), entropy (Δ_sol_*S*°), and Gibbs energy (Δ_sol_*G*°) change of solutes in water and 0.1 mol·kg^−^^1^ SS aqueous solutions were calculated from the results of experimental solubility data [26,27]:

(1) $${\left(\frac{\partial \ln x_{1}}{\partial \left(1/T-1/T_{\mathrm{hm}}\right)}\right)}_{p}=-\frac{\Delta_{\mathrm{sol}}H^{o}}{R}.$$

where *x*_1_ is the mole fraction of caffeine in solutions, *T* is the absolute temperature (K), and *R* is the universal gas constant with a value of 8.314 J·K^−^^1^·mol^−^^1^. *T*_hm_ represents the harmonic temperature, defined as:

(2) $$T_{\mathrm{hm}}=n/\sum_{j-1}^{n}\frac{1}{T_{j}},$$

where *n* equals the number of temperature points, *T*_j_ is the experimental temperature, and the obtained value is 302.99 K. Δ_sol_*H*° is defined as the apparent standard mole dissolution enthalpy change of solute dissolved in water and 0.1 mol∙kg^−^^1^ SS aqueous solution, obtained from the slope of the fitted line curves, ln*x*_1_ versus (1/*T* − 1/*T*_hm_) (Figure 3).

From the intercept obtained from the modified Van ’t Hoff plot, the Δ_sol_*G*° and Δ_sol_*S*° can be calculated by the following equations:

(3) $$\Delta_{\mathrm{sol}}G^{o}=-RT_{\mathrm{hm}}Intercept,$$

(4) $$\Delta_{\mathrm{sol}}S^{o}=\frac{\Delta_{\mathrm{sol}}H^{o}-\Delta_{\mathrm{sol}}G^{o}}{T_{\mathrm{hm}}}.$$

Determination of the relative contribution of enthalpy (*ζ_H_*) and the relative contribution of entropy (*ζ_TS_*) were calculated using the following expressions [26,27]:

(5) $$\varsigma_{H=}\frac{\left|\Delta_{\mathrm{sol}}H^{o}\right|}{\left|\Delta_{\mathrm{sol}}H^{o}\right|+\left|T_{\mathrm{hm}}\Delta_{\mathrm{sol}}S^{o}\right|},$$

(6) $$\varsigma_{TS=}\frac{\left|T_{\mathrm{hm}}\Delta_{\mathrm{sol}}S^{o}\right|}{\left|\Delta_{\mathrm{sol}}H^{o}\right|+\left|T_{\mathrm{hm}}\Delta_{\mathrm{sol}}S^{o}\right|}.$$

The obtained apparent thermodynamic properties’ values of Δ_sol_*G*°, Δ_sol_*H*°, and Δ_sol_*S°*, as well as of ζ*_H_* and *ζ_TS_* for caffeine in water and caffeine in SS aqueous solution, are presented in Table 2.

Table 2 shows that Δ_sol_*G*° and Δ_sol_*H*° values were positive for both examined solutions, caffeine in aqueous solution and caffeine in SS aqueous solution. The dissolution processes are endothermic, which indicates that dissolving caffeine in water and aqueous sodium salicylate solution proceeds with absorption of heat. A positive value of the enthalpy of dissolution indicates that the heat energy released due to the hydration of compounds is less than the energy required to break down the interactions between caffeine molecules. Based on these results, we can assume that in the presence of sodium salicylate, caffeine self-aggregates are formed in an aqueous solution, similar to that in pure water. Lower enthalpy values in sodium salicylate solution may be due to the hydration of sodium and salicylate ions. The negative Δ_sol_*S*° value for both examined solutions denotes that dissolution of caffeine in water proceeds with an increase in the order of the system. The formation of caffeine self-aggregates is undoubtedly one of the explanations for the negative values of dissolution entropy.

On the other hand, the influence of caffeine molecules on the structural organization of water molecules, i.e., its structure-making/breaking properties, will be examined based on volumetric and viscometric measurements. The calculated values of the relative contribution of enthalpy and entropy imply that the main contributor to Δ_sol_*G*°, in caffeine dissolution in aqueous solutions and SS aqueous solutions, is enthalpy. Moreover, the endothermic enthalpy of the dissolution process is in accordance with the obtained solubility increase with rising temperature.

### 3.2. Volumetric Properties

For the determination of caffeine volumetric properties in 0.1 mol·kg^−1^ SS aqueous solution, data obtained from experimental measurements of solution densities were collected and provided in Table S2. Densities of solutions increased with a molality of caffeine at all temperatures (Figure S1).

**Figure 4:**
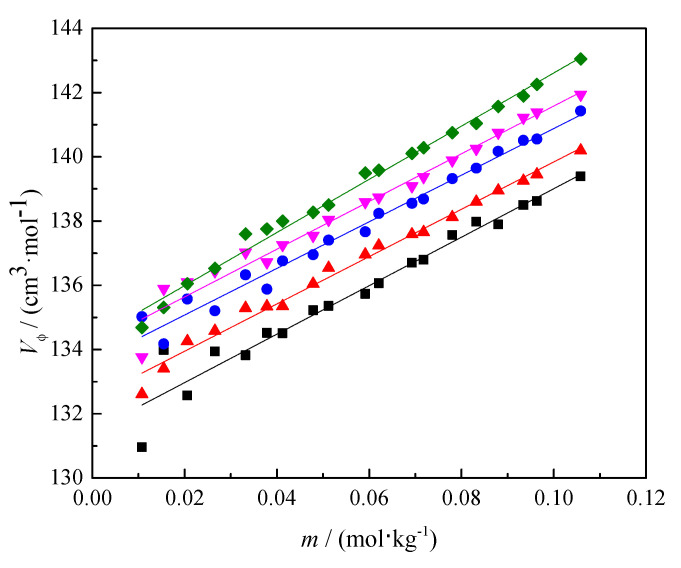
The dependence of the apparent molar volume ($V_{\phi }$) on caffeine molality (*m*) in caffeine in 0.1 mol∙kg^−1^ SS aqueous solutions at different temperatures (*T*) = (■) 293.15, (▲) 298.15, (●) 303.15, (▼) 308.15, and (◆) 313.15 K.

The obtained solution densities were used to calculate the apparent molar volume ($V_{\phi }$) of caffeine, from the equation presented in the Supplementary Materials. The calculated values are shown in Table S2. As shown in Figure 4, the caffeine values in 0.1 mol∙kg^−1^ SS aqueous solutions increased with caffeine molality at all temperatures.

The $V_{\phi }$ values were fitted with the Masson’s equation modified for non-electrolytes [28]:

(7) $$V_{\phi }=V_{{}_{\phi }}^{o}+S_{v}\cdot m,$$

and the plots of $V_{\phi }$ versus m presented in Figure 3. The $V_{\phi }^{o}$ value represents the apparent molar volume of solute at infinite dilution, and S*_v_* is the experimental slope, which provides information about solute–solute interactions. The obtained values of $V_{\phi }^{o}$ and S*_v_* are shown in Table 3 with the standard deviation (*σ*) and regression coefficient (*R*^2^).

The positive *S_v_* values indicate the existence of strong caffeine–caffeine interactions. These values are significantly higher than pure water and 0.1 mol∙kg^−1^ ATP aqueous solution (see Table S3). As caffeine self-aggregation occurs in pure water and 0.1 mol∙kg^−1^ ATP water solution [12], higher values of the *S_v_* coefficient indicate that the process of self-aggregation in the presence of SS is even more pronounced. At first glance, this is unusual. The self-aggregation of molecules usually reduces their solubility in water, while our results showed an increase in solubility of caffeine in the presence of sodium salicylate.

Furthermore, the obtained $V_{\phi }^{o}$ values of caffeine in SS aqueous solutions were positive and increased with rising temperature (Figure S2), but they were significantly lower than in pure water or in the presence of ATP molecules (see Table S3). This means that adding caffeine to a SS aqueous solution will lead to a significantly smaller increase in volume than in pure water or in the presence of ATP. Lower values of apparent molar volumes may also be a consequence of enhanced self-aggregation of caffeine molecules in the presence of sodium salicylate and/or strong interactions between caffeine molecules and salicylate anion. It is not possible to draw a conclusion based on volumetric measurements alone. That is why we need viscosity measurements and computer simulations.

The values of $V_{\phi }^{o}$ were fitted as a function of the temperature using the equation of the second order:

(8) $$V_{\phi }^{o}=a_{0}+a_{1}T+a_{2}T^{2}.$$

Calculated coefficients *a*_0_, *a*_1_, and *a*_2_ of caffeine in 0.1 mol∙kg^−1^ SS aqueous solutions together with regression coefficients (*R*^2^) are presented in Table S4. Based on the calculated coefficients, the limiting apparent molar expansibility, $E_{\phi }^{o}$, of caffeine in SS aqueous solutions was calculated and presented in Table 4, together with values in pure water:

(9) $$E_{\phi }^{o}={\left(\frac{\partial V_{\phi }^{o}}{\partial T}\right)}_{p}=a_{1}+2a_{2}T.$$

The values of $E_{\phi }^{o}$ were positive in the whole temperature range and significantly higher at lower temperatures than in pure water (Table 4 and Figure S3). Higher $E_{\phi }^{o}$ values indicate a faster release of water molecules from the hydration sphere. However, $E_{\phi }^{o}$ values decreased much faster with increasing temperature in the presence of SS compared to pure water. The Hepler’s coefficient defines the rate of this change and can be calculated with the following equation [29]:

(10) $${\left(\frac{\partial E_{\phi }^{o}}{\partial T}\right)}_{p}={\left(\frac{\partial^{2}V_{\phi }^{o}}{\partial T^{2}}\right)}_{p}=2a_{2}.$$

The obtained negative values for Hepler’s coefficient, presented in Table 4, suggest caffeine structure-breaking properties in sodium salicylate aqueous solutions. However, the efficiency of Hepler’s coefficient to estimate the structure-making/breaking properties in water systems where significant self-aggregation of molecules occurs is quite questionable.

From the available data for $V_{\phi }^{o}$ of caffeine in water and SS aqueous solutions, the limiting apparent transfer molar volumes ($\Delta_{tr}V_{\phi }^{o}$) can be calculated with the following equation [12]:

(11) $$\Delta_{\mathrm{tr}}V_{\phi }^{o}=V_{\phi }^{o}(\mathrm{caffeine}+0.1\mathrm{mol}\cdot {\mathrm{kg}}^{-1}\mathrm{aqueous}\;\mathrm{SS}\;\mathrm{solution})-V_{\phi }^{o}(\mathrm{caffeine}+\mathrm{water}).$$

Values of $V_{\phi }^{o}$ for the aqueous solution of caffeine are presented in Table S3. The obtained values are presented in Table 5. The negative values of $\Delta_{tr}V_{\phi }^{o}$ indicate a more pronounced self-aggregation of caffeine in the presence of SS or a strong interaction between caffeine and SS, which was discussed earlier in this paper.

According to Birch et al. [30], an organization of water molecules around the solute regulates transport in the taste epithelium, binding to the receptor and finally inducing a taste response. In this study, to define the taste quality of studied solutes, the apparent specific volume at infinite dilution, $V_{\phi }^{o}$ (results in Table 5), was calculated by dividing caffeine $V_{\phi }^{o}$ values with caffeine molar mass (*M* = 194.19 g·mol^−1^). It was found that the apparent specific volume at infinite dilution is a parameter of taste quality in the following order: salty (0.1–0.3 cm^3^·g^−1^) < sour (0.3–0.5 cm^3^·g^−1^) < sweet (0.5–0.7 cm^3^·g^−1^) < bitter (0.7–0.9 cm^3^·g^−1^). From Table 5, it can be seen that the calculated values of $V_{\phi }^{o}\approx$ 0.69 cm^3^·g^−1^ are on the border between the sweet and bitter taste of caffeine in SS aqueous solutions at all examined temperatures. On the other hand, Vraneš et al. calculated values for caffeine in pure water, 0.73–0.75 cm^3^·g^−1^, pointing out the bitter taste of caffeine at all examined temperatures (283.15–313.15 K) [12]. Thus, the presence of SS in water reduces the bitter taste of caffeine. These data are essential due to the joint presence of caffeine and benzoic acid derivatives in food products.

### 3.3. Viscosimetric Properties

The viscosity measurements of caffeine in 0.1 mol∙kg^−1^ SS aqueous solutions were measured in the temperature range from *T* = 293.15 to 313.15 K up to molality of caffeine *m* = 0.1058 mol·kg^−1^ at the atmospheric pressure (p = 1 × 10^5^ Pa). Data obtained from experimental caffeine measurements in 0.1 mol∙kg^−1^ SS aqueous solution viscosities are presented in Table S5, together with the values for aqueous solutions of caffeine [12]. Table S5 shows viscosity values for 0.1 mol∙kg^−1^ SS in water. Viscosity values of 0.1 mol∙kg^−1^ SS in water were higher than viscosities of pure water.

**Figure 5:**
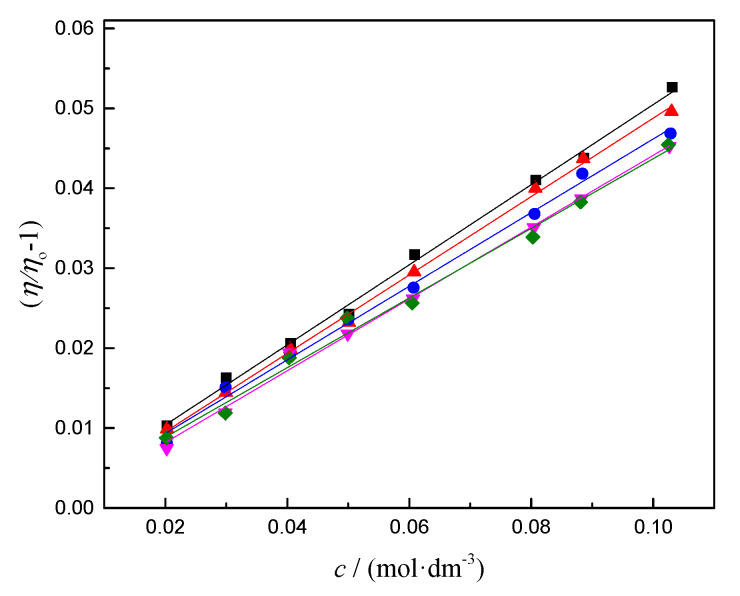
Plot of reduced viscosity (*η*/*η*_o_ − 1) versus concentration (*c*) of the caffeine in SS aqueous solutions, at different temperatures, *T* = (■) 293.15, (▲) 298.15, (●) 303.15, (▼) 308.15, and (◆) 313.15 K.

The viscosity values for caffeine in 0.1 mol∙kg^−1^ SS aqueous solution were higher than the values in pure water. In both systems, viscosity decreased with the temperature increase. One of the most common criteria for the determination of water organization around solute is the viscosity *B*-coefficient obtained from the Jones–Dole’s equation [31]:

(12) $$\frac{\eta }{\eta_{o}}-1=Bc,$$

where *η*_o_ is the viscosity of the pure solvent, *η* is the viscosity of the caffeine in SS aqueous solutions, and *c* is concentration. Following the Jones–Dole’s equation, the reduced viscosity (*η*/*η*_o_) dependence on the concentration was linear (Figure 5). Calculated *B*-coefficients are presented in Table 6.

The relation between the *B*-coefficient from the Jones–Dole’s equation and the change in the structural order of water molecules after adding a solute is well-known. If values of the *B*-coefficient are positive, that indicates the structure-making properties of the solute, pointing out an increase in the local order of the water molecules. Negative *B*-coefficient values indicate structure-breaking properties, weakening hydrogen bonds between water molecules near the solute [32]. From the calculated results given in Table 6, *B*-coefficients for caffeine in SS aqueous solutions had positive values and decreased with the rising temperature. Thus, both conditions (*B* > 0 and d*B*/d*T* < 0) were satisfied, and the caffeine molecule in 0.1 mol∙kg^−1^ sodium salicylate water solution can be declared a structure-maker. Table 6 also includes the *B*-coefficients of caffeine in aqueous solutions tested by Vraneš et al. [12]. From Table 6, it can be seen that *B*-coefficients in pure water were positive and increased with the rising temperature, suggesting that caffeine is an atypical structure-maker. The values of the *B*-coefficient in the presence of sodium salicylate were higher compared to pure water, especially at lower temperatures. At higher temperatures (313.15 K), these values were similar in both systems. The obtained trends indicate that the influence of caffeine on the local arrangement of water molecules at higher temperatures is similar and that the presence of SS makes a significant difference only at lower temperatures. After analyzing the results of volumetric and viscometric measurements, we can conclude that the presence of SS promotes the self-aggregation of caffeine in water. The formation of self-aggregates contributes to the structural arrangement of water. However, it remains unclear what interactions occur between caffeine molecules and sodium salicylate and whether salicylate anion is incorporated into aggregates.

### 3.4. Thermodynamic Activation Parameters for Viscous Flow

Feakins et al. [33] suggested calculation of the transition-state treatment of relative viscosity using viscosity obtained data:

(13) $$\Delta \mu_{1}{}^{o\neq }=RT\ln\left(\frac{\eta_{o}{\bar{V}}_{1}^{o}}{hN_{A}}\right)$$

where $\Delta \mu_{1}^{o\neq }\;$ is free energy of activation of viscous flow per mole of the pure water, $N_{A}$ is Avogadro’s number, ${\bar{V}}_{1}^{o}$ is partial molar volumes of water at infinite dilution, *h* is the Planck constant, *η*_o_ is the viscosity of the solvent, *R* is the universal gas constant, and *T* is the absolute temperature. The following equation calculates the free energy of activation of viscous flow per mole of solute:

(14) $$\Delta \mu_{2}{}^{o\neq }=\Delta \mu_{1}{}^{o\neq }+\frac{RT}{{\bar{V}}_{1}^{o}}\left(B+{\bar{V}}_{2}^{o}-{\bar{V}}_{1}^{o}\right),$$

where *B* is the viscosity coefficient, and ${\bar{V}}_{2}^{o}$ is the partial molar volume at infinite dilution of solute. The calculated values of ${\bar{V}}_{1}^{o}$, ${\bar{V}}_{2}^{o}$, $\Delta \mu_{1}^{o\neq }$, and $\Delta \mu_{2}^{o\neq }$ are presented in Table 7.

The values of $\Delta \mu_{2}^{o\neq }$ were positive and larger than $\Delta \mu_{1}^{o\neq }$ at all investigated temperatures, indicating that the interactions between caffeine and solvent molecules (water and SS) are more pronounced in the ground state than in the transition state. The transition state formation occurs with the breaking and deformation of the intermolecular bonds between solvent molecules [34,35]. The positive values of the term ($\Delta \mu_{2}^{o\neq }-\Delta \mu_{1}^{o\neq }\;)$ were described by Glasstone et al. as “loss of the structure-breaking contribution special to liquid water” [36]. In other words, the positive values of a mentioned term are typical for structure-making compounds. These data agree well with the analyzed results of the Jones-Dole’s equation’s *B*-coefficient.

The equation for determination of the entropy of activation for the viscous flow ($\Delta S_{2}^{o\neq }$) of the caffeine in 0.1 mol∙kg^−1^ SS aqueous solutions is:

(15) $$\Delta S_{2}{}^{o\neq }=-\frac{\partial \left(\Delta \mu_{2}{}^{o\neq }\right)}{\partial T},$$

while the activation enthalpy ($\Delta H_{2}^{o\neq }$) can be calculated with the equation:

(16) $$\Delta H_{2}^{o\neq }=\Delta \mu_{2}{}^{o\neq }+T\Delta S_{2}{}^{o\neq }.$$

The values of $\Delta S_{2}^{o\neq }$ and $\Delta H_{2}^{o\neq }$ were 239.66 J·mol^−1^·K^−1^ and 162.33 kJ·mol^−1^ for caffeine in SS aqueous solutions. These results support that formation of the transition state of caffeine in SS aqueous solutions is associated with bond-breaking and a decrease in order [34,35].

### 3.5. Hydration Number

The results of volumetric and viscosimetric measurements and higher solubility of caffeine indicate more significant caffeine interactions with water molecules in the presence of SS. Therefore, it is necessary to calculate the hydration number of caffeine in 0.1 mol∙kg^−1^ sodium salicylate water solution. The hydration number of caffeine is calculated from the volumetric properties using the following equation [37]:

(17) $$h_{n}=\frac{V_{\phi }^{o}\left(e\right)}{V_{e}^{o}-V_{b}^{o}},$$

where $V_{\phi }^{o}\left(e\right)$ is the electrostriction partial molar volume, $V_{e}^{o}$ is the molar volume of electrostricted water, and $V_{b}^{o}$ is the molar volume of bulk water. The literature values of $\left(V_{e}^{o}-V_{b}^{o}\right)$ are presented in Table S6. The mathematical approach for calculating the hydration number from the volumetric and viscosimetric parameters is described in the Supplementary Materials. The hydration numbers calculated in both described ways for caffeine in 0.1 mol∙kg^−1^ SS aqueous solution are presented in Table 8, along with the data for pure water [12].

From Table 8, it can be seen that the hydration numbers of caffeine in 0.1 mol∙kg^−1^ SS aqueous solutions are in good correlation. The values of the hydration number decreased with temperature, which is in accordance with the results obtained from expansibility. In our previous study, we calculated the hydration numbers of caffeine in pure water (Table 8), and the values obtained were significantly lower than those obtained in the presence of SS. However, these values were obtained at significantly lower concentrations of caffeine (about 0.06 mol∙kg^−1^), at which self-aggregation does not occur. To better understand the hydrating properties of caffeine at higher concentrations and the role of SS in increasing caffeine solubility, we will look for the answer in computer simulations.

### 3.6. Computational Study

Computational studies were used to confirm the results obtained from volumetric, viscometric, and solubility measurements. MD simulations were performed at caffeine molality *m* = 0.25 mol∙kg^−1^ for both tested cases at a temperature 298.15 K (250 molecules of caffeine, 100 molecules of SS (0.1 mol∙kg^−1^), and 55,555 molecules of water, while in the case of caffeine in pure water, 250 molecules of caffeine and 55,555 molecules of water were used).

**Figure 6:**
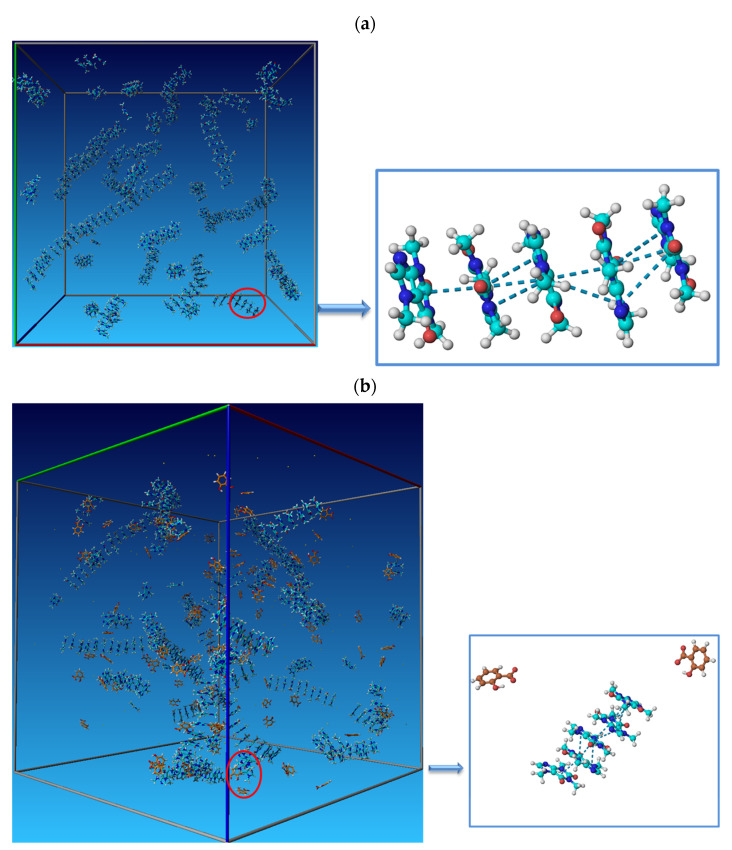
Visual representation obtained from the Yasara Structure program of caffeine organization in aqueous solution (**a**) and 0.1 mol∙kg^−1^ SS aqueous solution (**b**), and blue dashed lines represent π–π interactions between caffeine molecules.

**Figure 7:**
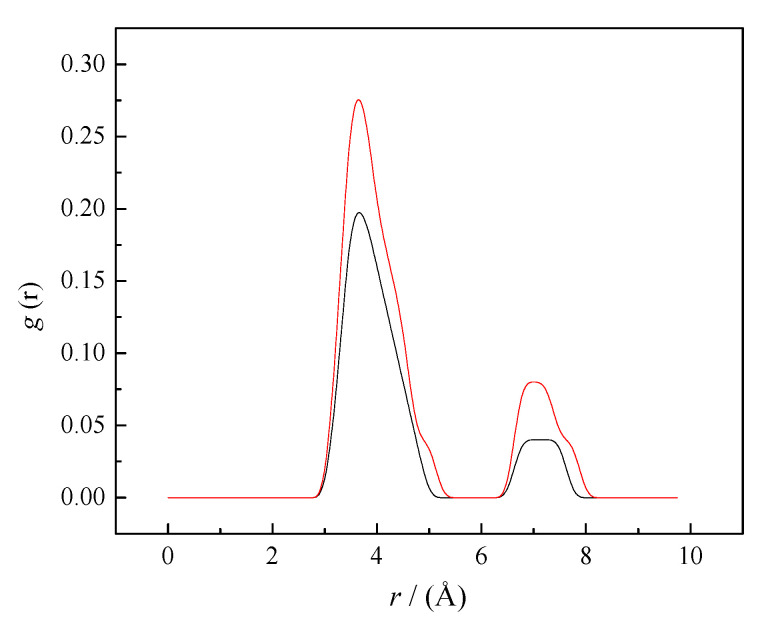
RDFs for the caffeine–caffeine center of mass (black line—aqueous solution, red line—0.1 mol∙kg^−1^ SS aqueous solution). The lines represent the radial distribution functions obtained from MD simulations’ trajectories.

It is a well-known scientific fact that caffeine molecules show a tendency towards self-aggregation in water solutions at higher concentrations [5,38]. As shown in Figure 6a, caffeine molecules tend to interact with each other, forming self-aggregates at molality *m* = 0.25 mol∙kg^−1^. Experimental results indicate that the presence of sodium salicylates increases the self-aggregation of caffeine molecules in water. Therefore, the radial distributions of caffeine molecules around the caffeine in pure water and the presence of SS were calculated. Figure 7 shows the obtained results from RDFs of the caffeine–caffeine center of mass. The first and second peaks of this distribution function appeared at 3.65 and 7.1 Å, indicating the existence of caffeine’s first and second coordination shells around caffeine.

**Figure 8:**
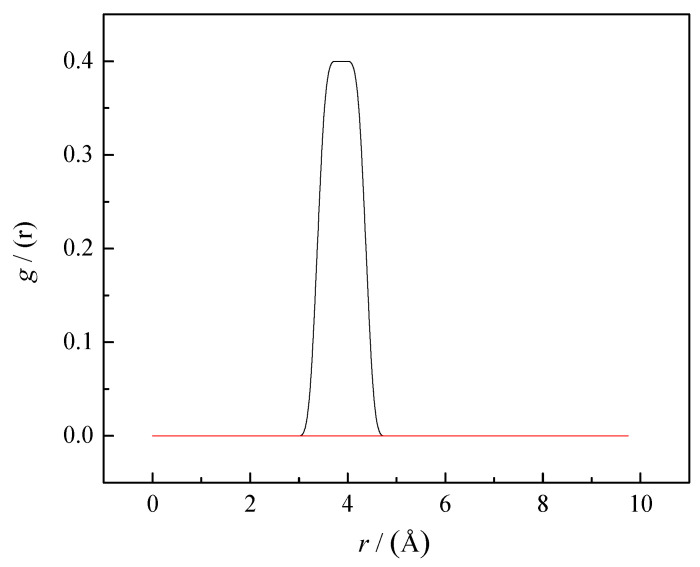
RDFs for caffeine-sodium salicylate center of mass. The lines represent the radial distribution functions obtained from MD simulations’ trajectories. The black line represents RDF for caffeine-sodium salicylate center of mass at 0.06 mol∙kg^−1^ and the red line represents RDF for caffeine-sodium salicylate center of mass at 0.25 mol∙kg^−1^.

The intensity of both peaks of radial function increased after the addition of SS to water, indicating increasing probabilities of self-aggregation between caffeine molecules. The large number of caffeine molecules in the coordination shells of caffeine confirmed that the presence of SS increased the self-aggregation of caffeine in water. Caffeine molecules interact through π–π interactions, as shown in Figure 6b. In Figure 6b, it can also be seen that salicylate molecules are not involved in caffeine self-aggregates. To confirm the absence of SS molecules from caffeine aggregates, RDF caffeine-salicylate was calculated, considering the center of masses of caffeine and salicylate. The calculated values were g(r) = 0 in the whole distance range from 0 to 10 Å, which confirms the absence of any interactions between these particles in equilibrium and at selected concentrations (Figure 8).

**Figure 9:**
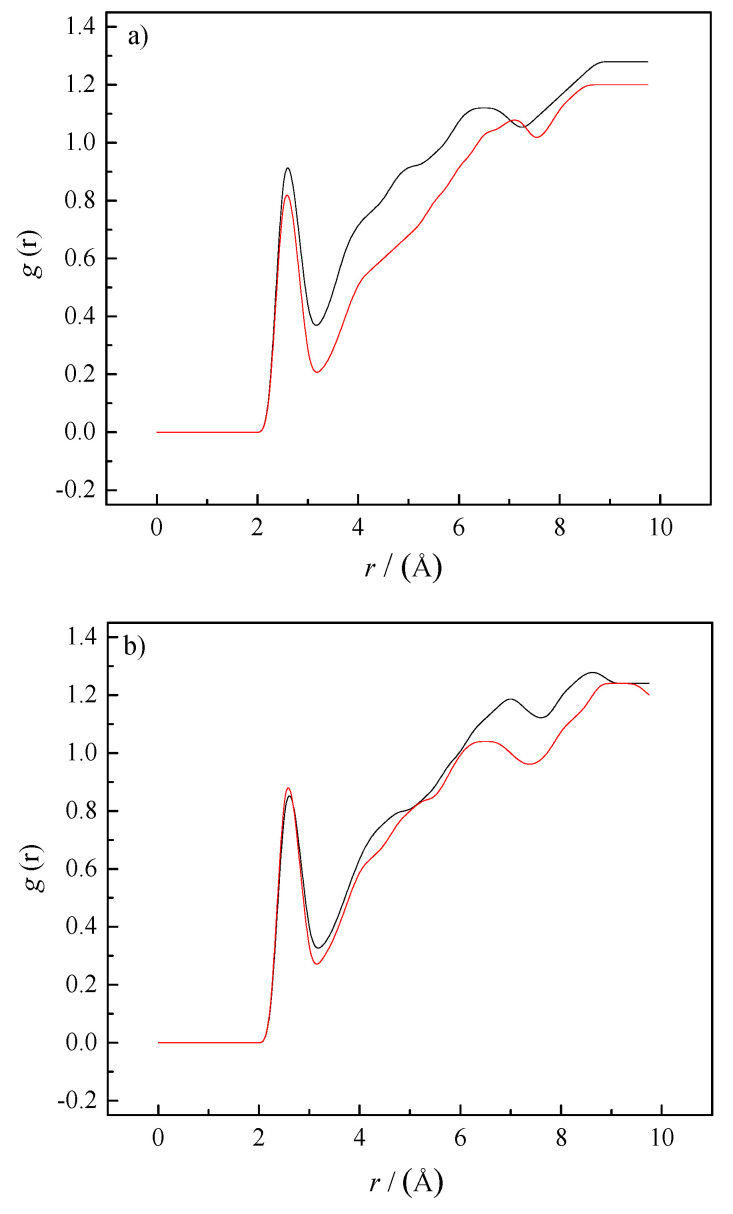

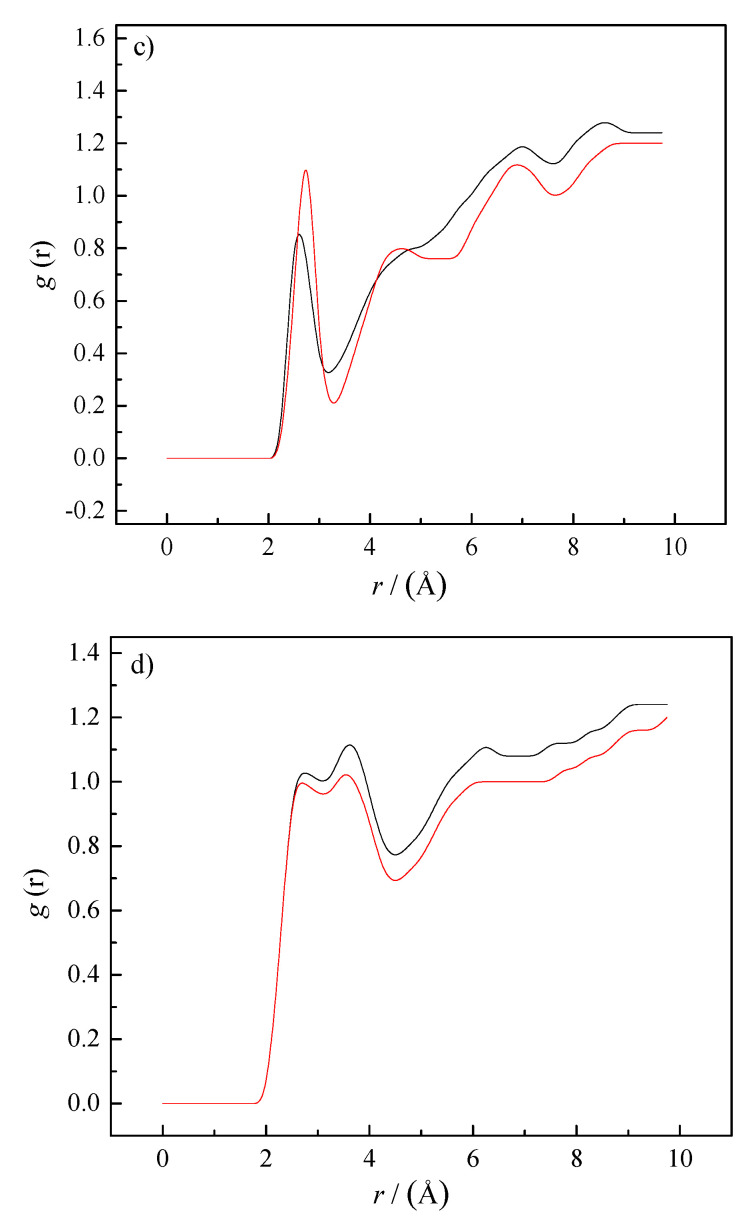
Radial distribution functions of water oxygen atom around selected atoms of caffeine in aqueous solution (black lines) and the presence of SS (red lines): (**a**) O1-Ow, (**b**) O2-Ow, (**c**) N4-Ow, and (**d**) H1-Ow.

To further analyze the hydration of caffeine molecules in the presence and absence of SS, calculations of the radial distribution function (RDFs) were applied. Figure 9 shows the RDFs of water oxygen atoms (Ow) around selected atomic sites of caffeine: O1, O2, N4, and H1. Hydrophilic centers in the caffeine molecule have been selected because they can form the strongest interactions with water molecules. As hydrogen bond acceptors, oxygen atoms from carbonyl groups, O1 and O2, as well as N4 atom from the imidazole ring were selected. The most acidic hydrogen atom (H1) was also considered the potential proton-donor for forming the H-bond with water molecules. Figure 9a–d show the values of g(r) in pure water and in the presence of sodium salicylate for all selected caffeine atoms. The obtained results indicate that sodium salicylate reduces the number of water molecules in the hydration spheres of O1 and H1 atoms. In the hydration sphere of the imidazole nitrogen atom (N4), the number of water molecules increased in the presence of SS, while in the case of carbonyl oxygen (O2), it did not change.

According to the procedure published elsewhere [12], the hydration number of caffeine in the presence and absence of SS was calculated to quantify the obtained results. The calculated value of the hydration number of caffeine in pure water at 298.15 K and caffeine molality *m* = 0.25 mol∙kg^−1^ was *h_n_* = 5.25. In 0.1 mol∙kg^−1^ sodium salicylate aqueous solutions, at the same molality, the hydration number of caffeine had a lower value, *h_n_* = 4.24. This value of hydration number in the presence of SS is in good agreement with the experimentally obtained results (Table 8). In our previous work [12], the hydration number of caffeine at a molality of 0.06 mol∙kg^−1^ (unfortunately, these data are missing in that paper) was calculated using an identical computer simulations procedure, and a value of *h_n_* = 0.82 at *T* = 298.15 K was obtained. At a molality of 0.06 mol∙kg^−1^, self-aggregation of caffeine in water was not observed. Thus, computer simulations show that the caffeine hydration number increased significantly during self-aggregation (from 0.82 to 5.25). The main reason for the increase in hydration number is that caffeine molecules form aggregates primarily through π–π interactions (see Figure 6b). As a result, caffeine molecules’ hydrophobic surfaces in self-aggregate are reduced [38], while hydrophilic centers have more probability for interaction with water molecules.

The presence of SS reduces the hydration number of caffeine (from 5.25 to 4.24), which is in concordance with the results of other studies. Sodium salicylate and many other polar molecules, such as sodium chloride [38], ATP [12], and sucrose [5], have a dehydrating effect on caffeine molecules. The mentioned molecules have more pronounced hydration abilities than caffeine, and by binding water molecules to themselves, they reduce the number of water molecules in the hydration shells of caffeine.

However, the main question remains open: how does sodium salicylate increase the solubility of caffeine in water? If we compare the results of the molecular dynamics simulations for the system NaCl + caffeine with our results, we can conclude that they are very similar [38]. In the presence of NaCl, caffeine self-aggregation in water increases, and RDFs peaks occur at the exact distances from the caffeine–caffeine center of mass. The addition of NaCl reduces the hydration number of water molecules around the hydrophilic centers of caffeine. These results were also obtained in this paper. Furthermore, both NaCl and SS have structure-making properties to water molecules.

The work of Ahmad et al. [7] showed that the presence of a cosmotrope (structure-makers, such as NaCl and Na_2_SO_4_) increases self-aggregation and decreases caffeine solubility, which is a consequence of the salting-out effect of these salts. The salting-out effect on caffeine molecules is also shown by some neutral molecules, such as sucrose [5]. Sucrose has structure-making properties, increasing the self-aggregation of caffeine and reducing its solubility.

However, SS has structure-maker properties but increases the solubility of caffeine.

The cosmotropic behavior of some additives does not appear to be a “driving force” to promote caffeine self-aggregation in water. Combining the Kirkwood–Buff solution theory with the isodesmic model of caffeine association, Shimizu [39] concluded that changes in the water structure upon the addition of cosmotropic or chaotropic additives have a negligible effect on the self-aggregation of caffeine. In the same paper, Shimizu concludes that the real driving force for caffeine self-aggregation is the interaction between additives and caffeine. Self-aggregation increases if additives are removed from caffeine molecules and decreases if additives stick around caffeine. In the mentioned manuscript, the author emphasizes that a new theory has important consequences for predicting the influence of additives on the solubility of macromolecules, but he did not elaborate on that influence.

**Figure 10:**
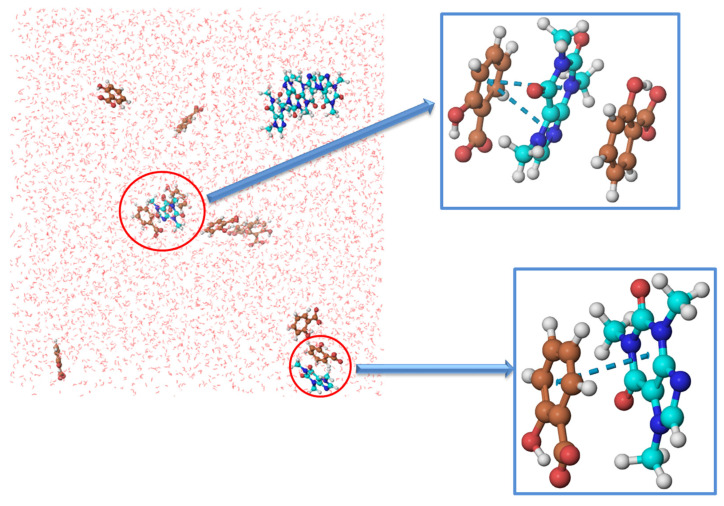
Visual representation obtained from the Yasara Structure program of caffeine organization in 0.1 mol∙kg^−1^ SS aqueous solution, and blue dashed lines represent π–π interactions. The molal concentration of caffeine is 0.06 mol∙kg^−1^.

Therefore, we need to analyze what interactions SS can form with the caffeine molecule, increasing its solubility, unlike sodium chloride and sucrose. The logical answer is π–π interactions because both salicylate and caffeine are aromatic compounds. However, we have already discussed (Figure 6b) that salicylate anions are not included in caffeine self-aggregates. Therefore, we set up a new MD simulation with a significantly lower concentration of caffeine (0.06 mol∙kg^−1^). At this concentration, self-association in pure water does not occur. The concentration of SS was 0.1 mol∙kg^−1^ and the number of water molecules was 5555. Figure 10 shows a snapshot of the distribution of caffeine and SS molecules after equilibrium (30 ns). It can be seen from the figure that the presence of SS initiated the self-aggregation of caffeine, but that not all molecules are included in the aggregates. However, Figure 10 also shows π–π interactions between caffeine and salicylate molecules. We calculated the radial distribution function concerning the center of masses to better understand the spatial relationship of salicylate and caffeine molecules (see Figure 8). The peak maximum appeared at a distance of 3.86 Å, which is very close to the value of the first peak of the radial distribution of the caffeine–caffeine center of mass (3.65 Å). This means that a molecule of caffeine displaces salicylate during aggregation and takes its place next to another molecule of caffeine. Finally, we can assume the mechanism of salicylate increasing caffeine’s solubility.

**Figure 11:**
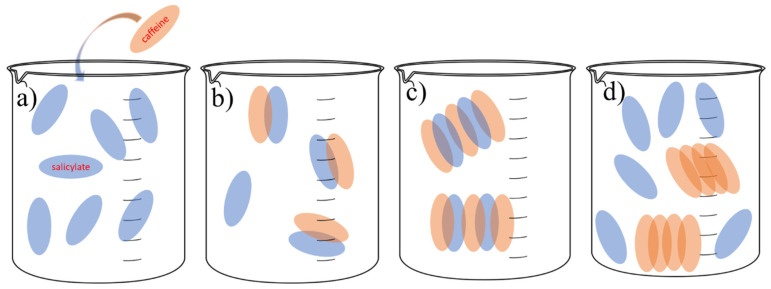
Visual representation of the potential role of salicylates in increasing the solubility of caffeine: (**a**) adding caffeine in SS solution, (**b**) forming π–π interactions between caffeine and salicylate anions, (**c**) caffeine and salicylate complexes that provide better solubility of caffeine monomers, and (**d**) forming caffeine–caffeine complexes through π–π interactions with salicylate anion release.

After adding caffeine to the SS solution (Figure 11a), a parallel stacking (π–π) aromatic complex between aromatic rings was formed (Figure 11b). Caffeine-salicylate complexes provide better solubility of caffeine monomers and allow local caffeine supersaturation formation (Figure 10c). After contact with two or more complexes, the salicylate anion was released, forming a π–π interaction between caffeine molecules (Figure 11d). The driving force for this process is the good solvation of the salicylate anion by water molecules and the tendency of caffeine molecules to aggregate with each other. We calculated the hydration number for salicylate anions not bonded with caffeine (see Figure 6b), and the average value was 5.47. Hydration of the salicylate ion is an exothermic process. Additional heat release due to hydration of the salicylate anion is a reason why the enthalpy of dissolution is lower in the presence of SS than in pure water. Releasing salicylate molecules from the complex with caffeine and its hydration after that is probably the main reason for increasing the solubility of caffeine in water.

Therefore, for an additive to increase caffeine’s solubility in water through forming caffeine self-aggregates, it needs to form π–π interactions with caffeine and have pronounced hydrating properties. This hypothesis needs to be tested in the presence of several additives. It is necessary to choose additives that can and cannot form π–π interactions with caffeine and additives with different hydrating properties, i.e., structure-makers and structure-breakers.

## 4. Conclusions

The objective of this study was to investigate the solubility of caffeine in the presence of SS both experimentally and computationally. The solubility of caffeine was 2–2.7 times higher in 0.1 mol∙kg^−1^ SS aqueous solutions than in pure water, and values increased with the rising temperature. Obtained results from volumetric and viscosimetric measurements indicated that SS increased caffeine self-aggregation in water and significantly reduced the bitterness of caffeine molecule in aqueous solution. The hydration number of caffeine increased after self-aggregation. The increase in caffeine solubility in the presence of salicylates is not a consequence of the structure-making properties of the salicylate anion but its interactions with caffeine molecules. The mechanism by which salicylate affects the solubility of caffeine in an aqueous solution has been hypothesized. The better solubility of caffeine monomers and the ability to achieve local supersaturation of solution are probably consequences of forming complexes with the salicylate anion through π–π interactions. Release and hydration of the salicylate anion after self-aggregation of caffeine is the main reason for the lower enthalpy dissolution of caffeine in the presence of SS. It remains an open question whether self-aggregation of caffeine always leads to a decrease in solubility. The authors know that it is not easy to answer these questions, so the plan is to start a series of experiments, for example, self-aggregation of caffeine in the presence of the ionic liquid based on benzoic acid derivates. One of them is the well-known ionic liquid 1-butyl-3-methylimidazolium salicylate, where we can assume that caffeine molecules can interact through pi–pi interactions with a cation and anion, or just one of them. The research conducted in this work is of great importance for future formulations in pharmacy, which will contain a combination of caffeine and sodium salicylate or only one of them.

## Figures and Tables

**Table 1:** Determined solubility of caffeine in water and caffeine in 0.1 mol∙kg^−^^1^ SS aqueous solutions in the temperature range from *T* = 291.15 to 313.15 K.

*T*/(K)	Caffeine Solubility/(g (1000 g_water_)^−1^)	
	Caffeine in Water	Caffeine in SS Aqueous Solution
293.15	16.33	43.83
298.15	20.71	49.26
303.15	25.29	55.73
308.15	34.83	68.62
313.15	43.90	89.22

**Table 2:** The obtained apparent thermodynamic values of Δ_sol_*G°*, Δ_sol_*H°*, and Δ_sol_*S°*, as well as ζ*_H_* and *ζ_TS_* for caffeine in water and SS aqueous solutions.

	Caffeine in Aqueous Solution	Caffeine in 0.1 mol∙kg^−1^ SS Aqueous Solution
Δ_sol_*G*°/(kJ·mol^−1^)	13.30	13.28
Δ_sol_*H*°/(kJ·mol^−1^)	0.41	0.13
Δ_sol_*S*°/(J·K^−1^·mol^−1^)	−0.0425	−0.0434
*ζ_H_*	0.031	0.009
*ζ_TS_*	0.96	0.99

**Table 3:** Fitting parameters obtained using the Masson′s equation modified for non-electrolytes of caffeine in SS aqueous solutions in the temperature range from *T* = 293.15 to 313.15 K, with the standard deviations (*σ*) and regression coefficients (*R*^2^).

*T*/(K)	$V_{{}_{\phi }}^{o}({\mathrm{cm}}^{3}\cdot \mathrm{mol}{-}^{1})$	*S_v_*/(cm^3^·kg·mol^−2^)	*σ* (cm^3^∙mol^−1^)	*R* ^2^
293.15	131.46	75.46	0.503	0.9497
298.15	132.47	73.66	0.234	0.9882
303.15	133.62	72.56	0.304	0.9796
308.15	134.15	74.28	0.375	0.9706
313.15	134.33	82.78	0.237	0.9903

**Table 4:** Values of the limiting apparent molar expansibilities, $E_{\phi }^{o}$, and Hepler’s coefficients of caffeine in SS aqueous solutions in the temperature range from *T* = 283.15 to 313.15 K.

T/(K)	293.15	298.15	303.15	308.15	313.15	${\left(\frac{\partial E_{\phi }^{o}}{\partial T}\right)}_{p}$/(cm^3^·mol^−1^·K^−2^)
	$E_{\phi }^{o}$/(cm^3^·mol^−1^·K^−1^)					
m(SS)/(mol·kg^−1^)						
0.0000	0.1690	0.1609	0.1528	0.1447	0.1366	−0.0016 [12]
0.1000	0.2791	0.2137	0.1484	0.0831	0.0178	−0.0130

**Table 5:** Limiting apparent transfer molar volumes, $\Delta_{\mathrm{tr}}v_{\phi }^{o}$, and the apparent specific volume at infinite dilution, $v_{\phi }^{o}$, of caffeine in 0.1 mol∙kg^−1^ SS aqueous solutions in the temperature range from T = 293.15 to 313.15 K.

*T*/(K)	$\Delta_{\mathrm{tr}}v_{\phi }^{o}$/(cm^3^·mol^−1^)	$v_{\phi }^{o}$/(cm^3^·g^−1^)
293.15	−11.47	0.68
298.15	−11.06	0.68
303.15	−10.83	0.69
308.15	−11.05	0.69
313.15	−11.58	0.69

**Table 6:** Values of viscosity *B*-coefficients obtained from the Jones-Dole’s equation for caffeine in aqueous solution [12] and in 0.1 mol∙kg^−1^ SS aqueous solutions in the temperature range from *T* = 293.15 to 313.15 K.

*T*/(K)	293.15	298.15	303.15	308.15	313.15
*m*(SS)*/*(mol·kg^−1^)	*B*/(dm^3^·mol^−1^)				
0.1000	0.501	0.490	0.461	0.449	0.436
0.0000 [11]	0.414	0.421	0.429	0.432	0.435

**Table 7:** Thermodynamic parameters ${\bar{V}}_{1}^{o}$, ${\bar{V}}_{2}^{o}$, $\Delta \mu_{1}^{o\neq }$, and $\Delta \mu_{2}^{o\neq }$ of viscous flow values of caffeine in 0.1 mol∙kg^−1^ SS aqueous solution in the temperature range from *T* = 293.15 to 313.15 K.

Parameters	*T*/(K)				
	293.15	298.15	303.15	308.15	313.15
${\bar{V}}_{2}^{o}$	131.46	132.47	133.62	134.15	134.33
${\bar{V}}_{1}^{o}$	18.18	18.21	18.23	18.26	18.30
$\Delta \mu_{1}^{o\neq }$	9.42	9.28	9.15	9.03	8.92
$\Delta \mu_{2}^{o\neq }$	91.77	91.62	88.78	88.31	87.43

**Table 8:** Values of caffeine hydration numbers gathered from volumetry and viscosimetric measurements in aqueous solution and 0.1 mol∙kg^−1^ SS aqueous solutions in the temperature range from *T* = 283.15 to 313.15 K.

*T*/(K)	*h_n_* (Volumetry)	*h_n_* (Viscosimetry)	*h_n_* (for Caffeine in Water, Volumetry [11])
293.15	4.23	4.17	0.94
298.15	3.67	3.28	0.68
303.15	3.04	2.58	0.38
308.15	2.61	2.17	0.16
313.15	2.23	1.39	−0.01

## Data Availability

Not applicable.

## Supplementary Materials

The following supporting information can be downloaded at: https://www.mdpi.com/article/10.3390/pharmaceutics14112304/s1, Figure S1: Variation of density (*d*) for caffeine in SS aqueous solutions as a function of molality, at different temperatures, (*T*) = (■) 293.15, (▲) 298.15, (●) 303.15, (▼) 308.15, and (◆) 313.15 K; Figure S2: Variation of the caffeine apparent molar volume at infinite dilution ( where $V_{\phi }^{o}$) with temperature in (■) SS aqueous solutions; Figure S3: Variation of the limiting apparent molar expansibility ( where $E_{\phi }^{o}$) of caffeine (▲) in SS aqueous solutions with temperature; Table S1: Provenance, purity, and structure of the used chemicals; Table S2: Molality (m), density (d), and apparent molar volume (*V*_φ_) of caffeine in 0.1 mol∙kg^−1^ SS aqueous solutions in the temperature range from *T* = 283.15 to 313.15 K at *p* = 1·105 Pa; Table S3: Fitting parameter obtained using the Masson′s equation modified for non-electrolytes of caffeine in aqueous solutions and in 0.1 ATP aqueous solutions in the temperature range from *T* = 293.15 to 313.15 K; Table S4: Fitting coefficients *a*_0_, *a*_1_, and *a*_2_ of caffeine in 0.1 mol∙kg^−1^ sodium salicylate aqueous solutions, with the regression coefficient (*R*^2^); Table S5: Viscosity of caffeine in aqueous solution [1] and in 0.1 mol∙kg^−1^ SS aqueous solutions in the temperature range from *T* = 283.15 to 313.15 K at *p* = 1·105 Pa; Table S6: The literature values of ($V_{e}^{o\;}-V_{b}^{o\;})$ in the temperature range from *T* = 293.15 to 313.15 K. References [12,40,41,42] are cited in the Supplementary Materials.
