# Supplementary material for: Influence of Sodium Salicylate on Self-Aggregation and Caffeine Solubility in Water—A New Hypothesis from Experimental and Computational Data
Source: Pharmaceutics. 2022 Oct 26;14(11):2304. doi: 10.3390/pharmaceutics14112304 (PMC9697389; doi:10.3390/pharmaceutics14112304)
Supplement: Supplementary file 1 [file pharmaceutics-14-02304-s001.zip › pharmaceutics-1935862-supplementary.pdf]

pharmaceutics

MDPI

Article

Supporting Information: Influence of Sodium Salicylate on Self-Aggregation and Caffeine Solubility in Water—A New Hypothesis from Experimental and Computational Data

Milan Vraneš [1], Teona Teodora Borović [1,*], Patrik Drid [2], Tatjana Trivić [2], Renato Tomaš [3] and Nenad Janković [4]

1 Faculty of Sciences, University of Novi Sad, Trg Dositeja Obradovića 3, 21000 Novi Sad, Serbia
2 Faculty of Sport and Physical Education, University of Novi Sad, Lovćenska 16, 21000 Novi Sad, Serbia
3 Faculty of Chemistry and Technology, University of Split, Ruđera Boškovića 35, HR-21000 Split, Croatia
4 Department of Chemistry, Faculty of Science, University of Kragujevac, Radoja Domanovića 12, 34000 Kragujevac, Serbia
* Correspondence: teona@dh.uns.ac.rs; Tel.: +381-21-485-2751

Density measurements. The densities of caffeine and aqueous sodium salicylate solutions were determined using a vibrating tube Rudolph Research Analytical DDM 2911 densimeter, and it was thermostated within ±0.01 K and viscosity was automatically corrected. Before each series of measurements, calibration of the instrument was performed at the atmospheric pressure (p = $1 \cdot 10^5$ Pa) using ambient air and bi-distilled ultra-pure water in the temperature range from T = (293.15 to 313.15) K. Each value of experimental density was obtained as the average of at least five measurements at selected temperatures. The repeatability of experimental measurements was within 0.01%, and the average value is presented in this paper. The standard uncertainty of determining the density is less than $4.31 \cdot 10^{-3}$g·cm^{-3}.

Viscosity measurements. Viscosities of caffeine and sodium salicylate aqueous solutions were determined by measuring the flow rate using *Ubbelohde* viscosimeter (SI Analytics GmbH, 523 03/0c). Calibration of the viscosimeter was done using 0.1 mol·dm^{-3} KCl solution (NIST reference) and bi-distilled deionized water in a temperature range from (293.15 to 313.15) K. Viscosimeter was filled with experimental solutions and placed vertically in the glass-sided thermostat maintained constant to ±0.01 K, with the standard uncertainty of controlled temperature of ±0.015 K. Calculation of the dynamic viscosity was obtained using the following equation:

$$\eta = d\left(Kt - \frac{L}{t}\right),$$

where K, L are constants of the viscosimeter (K = 68.1199 m^2·s^{-2}, L = $2.02 \cdot 10^{-2}$ m^2), t is a flow time and d experimental density of the liquid. The combined relative uncertainty of determining the viscosity with *Ubbelohde* viscosimeter was found to be less than 0.6%.

Table S1. Provenance, purity, and structure of the used chemicals.

Chemical Name	Source	CAS number	Mass fraction purity	Structure
Caffeine	Sigma Aldrich	58-08-2	≥99%	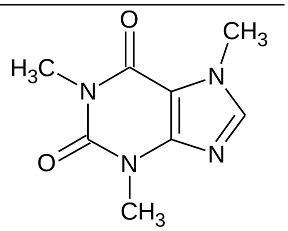
Sodium salicylate	Sigma Aldrich	54-21-7	≥99.5%	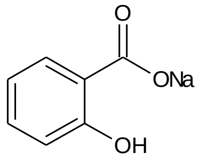
Chloroform	Lachner	67-66-3	≥99%	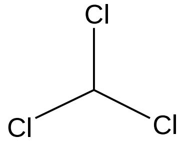

Table S2. Molality, (m), density, (d), and apparent molar volume, (V_Φ) of caffeine in aqueous in 0.1 mol·kg^{-1} sodium salicylate aqueous solutions in the temperature range from T = (283.15 to 313.15) K at p= 1·10^5 Pa.

m / (mol·kg^{-1})	d / (g·cm^{-3})					V_ϕ / (cm^3·mol^{-1})				
T / (K)	293.15	298.15	303.15	308.15	313.15	293.15	298.15	303.15	308.15	313.15
0.01076	1.00557	1.00427	1.00277	1.00113	0.99924	130.96	132.61	135.02	133.76	134.68
0.02061	1.00615	1.00484	1.00334	1.00168	0.99980	132.57	134.26	135.57	136.09	136.05
0.03321	1.00687	1.00555	1.00405	1.00237	1.00048	133.82	135.29	136.33	137.02	137.60
0.04124	1.00732	1.00601	1.00449	1.00282	1.00091	134.51	135.35	136.76	137.26	138.00
0.05123	1.00786	1.00653	1.00502	1.00334	1.00144	135.36	136.54	137.41	138.04	138.50
0.06208	1.00844	1.00710	1.00557	1.00389	1.00197	136.07	137.24	138.23	138.74	139.58
0.07180	1.00894	1.00761	1.00607	1.00438	1.00244	136.80	137.66	138.69	139.37	140.28
0.08322	1.00948	1.00816	1.00661	1.00492	1.00299	137.98	138.60	139.65	140.25	141.04
0.09343	1.00999	1.00865	1.00707	1.00536	1.00344	138.50	139.25	140.51	141.22	141.90
0.10584	1.01056	1.00921	1.00762	1.00593	1.00395	139.39	140.19	141.43	141.94	143.05
0.01549	1.00582	1.00455	1.00307	1.00138	0.99952	133.99	133.41	134.18	135.89	135.31
0.02657	1.00648	1.00518	1.00370	1.00201	1.00013	133.94	134.58	135.21	136.44	136.53
0.03784	1.00712	1.00582	1.00433	1.00264	1.00073	134.52	135.34	135.88	136.72	137.75
0.04785	1.00768	1.00636	1.00485	1.00317	1.00127	135.23	136.04	136.95	137.54	138.27
0.05915	1.00830	1.00695	1.00545	1.00374	1.00182	135.73	136.96	137.67	138.59	139.50
0.06928	1.00881	1.00747	1.00595	1.00426	1.00232	136.70	137.58	138.55	139.09	140.10
0.07803	1.00923	1.00792	1.00636	1.00467	1.00274	137.56	138.12	139.32	139.89	140.75
0.08797	1.00975	1.00838	1.00682	1.00512	1.00319	137.89	138.95	140.17	140.75	141.57
0.09636	1.01013	1.00879	1.00722	1.00550	1.00355	138.63	139.45	140.55	141.39	142.25

Standard uncertainties are: $u(d)$ = 3.04·10^{-4} g·cm^{-3}, $u(m)$ = 3.5·10^{-4} mol·kg^{-1}, $u(T)$ = 0.015 K, $u(V_\Phi)$ = 8.2·10^{-3} cm^3·mol^{-1}
Relative standard uncertainty: $u_r(p)$=0.015

From the experimental densities, the apparent molar volumes, V_ϕ , were calculated using the equation:

$$V_\phi = \frac{1000\left(d_2 - d\right)}{mdd_2} + \frac{M_1}{d},$$

where m (mol·kg^{-1}) is the molality of solute, d (g·cm^{-3}) is the experimental density of the solution, while d_2 is related to the solvent, and M_1 (g·mol^{-1}) is a molar mass of solute.

Table S3. Fitting parameter obtained using the *Masson*'s equation modified for nonelectrolytes, of caffeine in aqueous solutions and in 0.1 ATP aqueous solutions in the temperature range from T = (293.15 to 313.15) K [1].

T / (K)	V_ϕ^o / (cm^3·mol^{-1})	S_v / (cm^3·kg·mol^{-2})	σ	R^2
Caffeine aqueous solutions				
283.15	141.05	-10.44		0.9266
288.15	141.99	-12.34		0.9304
293.15	142.93	-14.85		0.9341
298.15	143.59	-13.60		0.9512
303.15	144.45	-15.35		0.9658
308.15	145.20	-15.69		0.9569
313.15	145.91	-15.46		0.9648
Caffeine in 0.1 ATP aqueous solutions				
283.15	142.24	18.22		0.9707
288.15	143.20	15.39		0.9348
293.15	144.20	10.68		0.9442
298.15	144.91	11.36		0.9326
303.15	145.81	8.763		0.9449
308.15	146.57	7.870		0.9646
313.15	147.38	6.523		0.9502

Table S4. Fitting coefficients a_0, a_1 and a_2 of caffeine in 0.1 mol·kg^{-1} sodium salicylate aqueous solutions, with the regression coefficient (R^2).

a_0 / (cm^3·mol^{-1})	a_1 / (cm^3·mol^{-1}·K^{-1})	a_2 / (cm^3·mol^{-1}·K^{-2})	R^2
-510.1	4.109	-0.0065	0.9869

Table S5. Viscosity of caffeine in aqueous solution [1] and in 0.1 mol·kg^{-1} sodium salicylate aqueous solutions in the temperature range from T = (283.15 to 313.15) K at p= 1·10^5 Pa.

m / (mol·kg^{-1})	η / (mPa·s)				
T / K	293.15	298.15	303.15	308.15	313.15
Caffeine aqueous solutions					
0.0201	1.007	0.892	0.799	0.720	0.652
0.0300	1.010	0.897	0.802	0.723	0.655
0.0400	1.013	0.901	0.805	0.725	0.657
0.0500	1.018	0.903	0.809	0.728	0.660
0.0599	1.022	0.907	0.812	0.732	0.663
0.0700	1.027	0.910	0.815	0.735	0.665
0.0800	1.031	0.916	0.819	0.738	0.669
Caffeine in 0.1 mol·kg^{-1}sodium salicylate aqueous solutions					
0.02061	1.056	0.935	0.834	0.747	0.677
0.03049	1.062	0.939	0.840	0.751	0.679
0.04124	1.066	0.944	0.843	0.756	0.684
0.05092	1.070	0.947	0.847	0.758	0.687
0.06208	1.078	0.953	0.850	0.761	0.688
0.08261	1.088	0.963	0.857	0.768	0.694
0.09103	1.091	0.966	0.862	0.771	0.697
Sodium salicylate in water					
0.1000	1.045	0.926	0.827	0.742	0.671

Calculation of hydration numbers: The hydration number were calculated from the volumetric and viscosimetric properties. The $V_\phi^o(e)$ is calculated from the experimentally obtained values V_ϕ^o:

$$V_\phi^o(e) = V_\phi^o - V_\phi^o(\text{int}) \quad \text{(S1)}$$

where $V_\phi^o(\text{int})$ is the intrinsic partial molar volume, calculated from:

$$V_\phi^o(\text{int}) = (0.7/0.634)V_\phi^o(\text{cryst}) \quad \text{(S2)}$$

where

$$V_\phi^o(\text{cryst}) = (M/d)(\text{cryst}) \quad \text{(S3)}$$

where d is the density of dry caffeine, with the value of 1.48 g·cm^{-3} and M is caffeine molar mass (M = 194.19 g·mol^{-1}) [2].

Calculating the hydration number (h_n) using the values of V_ϕ^o of caffeine and values of relative viscosity using the equation:

$$h_n = \frac{V_e^o - V_\phi^o}{V_{m,H_2O}}, \tag{S4}$$

where V_ϕ^o is the apparent molar volume at infinite dilution of caffeine in 0.1 mol·kg^{-1} sodium salicylate water solution, V_{m,H_2O} is the molar volume of water, V_e^o is the effective volume at infinite dilution of the flowing unit obtained by linear extrapolation of the V_e values against c to zero concentration of solute. The effective volume of the flowing unit (V_e) is calculated using *Breslau-Miller* equation (16) [3]:

$$V_e^o = \frac{(-2.5 \cdot c \cdot ((2.5 \cdot c)^2 - 4 \cdot (10.05 \cdot c^2)(1 - \frac{\eta}{\eta_o}))^{1/2})}{2 \cdot (10.50 \cdot c^2)} \tag{S5}$$

derived from the *Thomas* equation [3]:

$$\frac{\eta}{\eta_o} = 1 + 2.5 \cdot V_e \cdot c + 10.05 \cdot V_e^2 \cdot c^2 \tag{S6}$$

Table S6. The literature values of $(V_e^o - V_b^o)$ in the temperature range from T = (293.15 to 313.15) K [2].

T / (K)	$(V_e^o - V_b^o)$ / (cm^3·mol^{-1})
293.15	-3.1
298.15	-3.3
303.15	-3.6
308.15	-4.0
313.15	-4.4

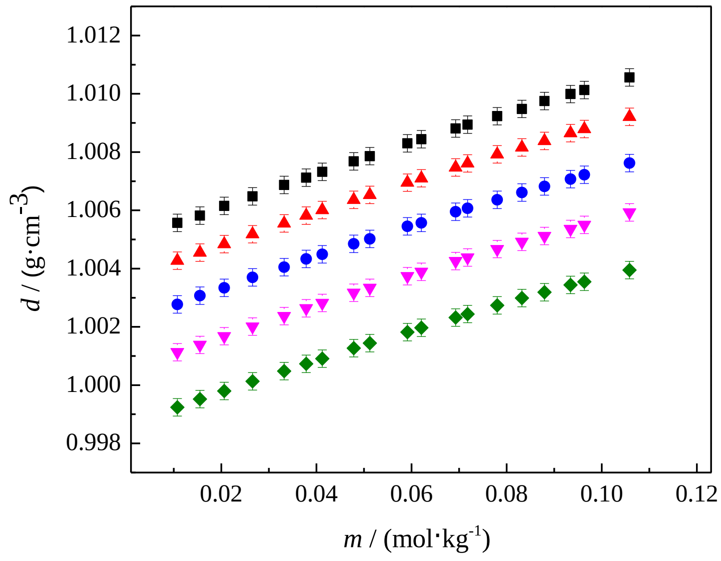

Figure S1.Variation of density, (*d*), for caffeine in sodium salicylate aqueous solutions as a function of molality, at different temperatures, (*T*): = (■) 293.15; (▲) 298.15; (●) 303.15; (▼) 308.15; (◆) 313.15 K.

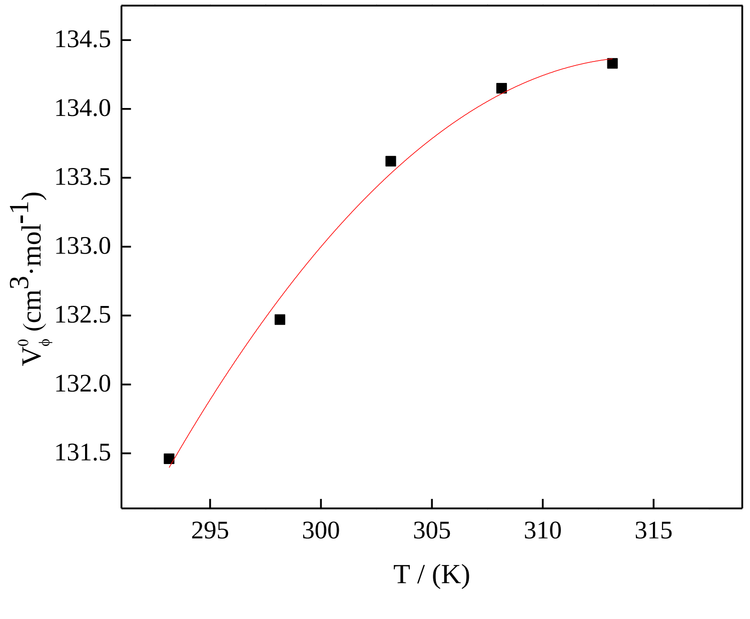

Figure S2. Variation of the caffeine apparent molar volume at infinite dilution (V_{ϕ}^{0}) with a temperature in (■) sodium salicylate aqueous solutions.

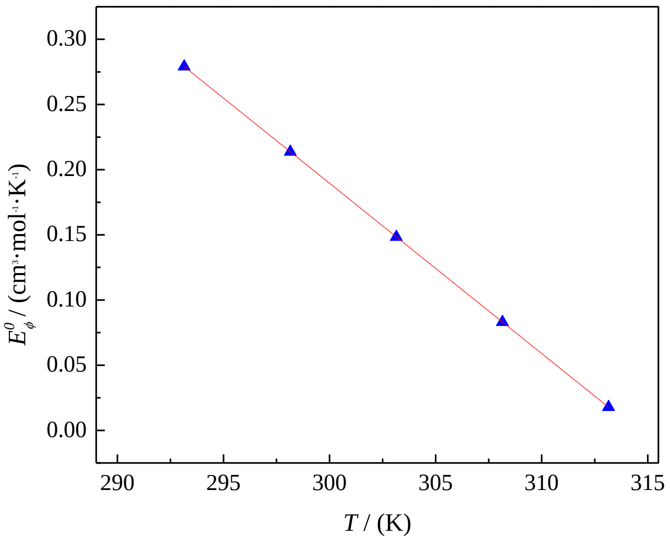

Figure S3. Variation of the limiting apparent molar expansibility, (E_ϕ^0) of caffeine (▲) in sodium salicylate aqueous solutions with a temperature.

References

1. Vraneš, M.; Panić, J.; Tot, A.; Gadžurić, S.; Podlipnik, Č., Bešter-Rogač, M., How the presence of ATP affect caffeine hydration and self-aggregation?. *J. Mol. Liq.* **2020,** 318, 113885.

2. Páez, M.; Figueredo, S.; Pérez, D.; Vergara, M.; Lans, E. Volumetric, viscometric and molecular simulation studies of glycine in aqueous sodium sulphate solutions at different temperatures. *J. Mol. Liq.* **2018,** 266, 718-726.

3. Begard, J.; Jadot, R. Solvation in aqueous *N,N*-dimethylformamide. *J. Chem. Thermodyn.* **1975**, 7, 1185–1188.

4. Thomas, D. G. Transport characteristics of suspension: VIII. A Note on the viscosity of Newtonian suspension of uniform spherical particles. *J. Colloid Sci.* **1965**, 20, 267–271.
